# Sensitive and Specific Fluorescent Probes for Functional Analysis of the Three Major Types of Mammalian ABC Transporters

**DOI:** 10.1371/journal.pone.0022429

**Published:** 2011-07-22

**Authors:** Irina V. Lebedeva, Praveen Pande, Wayne F. Patton

**Affiliations:** ENZO Life Sciences, Inc., Farmingdale, New York, United States of America; University of Illinois at Chicago, United States of America

## Abstract

An underlying mechanism for multi drug resistance (MDR) is up-regulation of the transmembrane ATP-binding cassette (ABC) transporter proteins. ABC transporters also determine the general fate and effect of pharmaceutical agents in the body. The three major types of ABC transporters are MDR1 (P-gp, P-glycoprotein, ABCB1), MRP1/2 (ABCC1/2) and BCRP/MXR (ABCG2) proteins. Flow cytometry (FCM) allows determination of the functional expression levels of ABC transporters in live cells, but most dyes used as indicators (rhodamine 123, DiOC_2_(3), calcein-AM) have limited applicability as they do not detect all three major types of ABC transporters. Dyes with broad coverage (such as doxorubicin, daunorubicin and mitoxantrone) lack sensitivity due to overall dimness and thus may yield a significant percentage of false negative results. We describe two novel fluorescent probes that are substrates for all three common types of ABC transporters and can serve as indicators of MDR in flow cytometry assays using live cells. The probes exhibit fast internalization, favorable uptake/efflux kinetics and high sensitivity of MDR detection, as established by multidrug resistance activity factor (MAF) values and Kolmogorov-Smirnov statistical analysis. Used in combination with general or specific inhibitors of ABC transporters, both dyes readily identify functional efflux and are capable of detecting small levels of efflux as well as defining the type of multidrug resistance. The assay can be applied to the screening of putative modulators of ABC transporters, facilitating rapid, reproducible, specific and relatively simple functional detection of ABC transporter activity, and ready implementation on widely available instruments.

## Introduction

Multidrug resistance relates to resistance of tumor cells to a whole range of chemotherapy drugs with different structures and cellular targets [1]. The phenomenon of multi drug resistance (MDR) is a well known problem in oncology and thus warrants profound consideration in therapeutic treatment of cancer. One of the underlying molecular mechanisms responsible for MDR is the up-regulation of a family of MDR transporter proteins that lead to chemotherapy resistance in cancer by actively extruding a wide variety of therapeutic compounds from the malignant cells. MDR transporters belong to an evolutionarily conserved family of ATP binding cassette (ABC) proteins, expressed in practically all living organisms from prokaryotes to mammals [2]. The same ABC transporters play an important protective function against toxic compounds in a variety of cells and tissues, especially in secretory organs, at the sites of absorption, and at blood-tissue barriers. The three major multidrug resistance ABC proteins are MDR1 (P-glycoprotein, ABCB1), multidrug resistance associated protein 1 (MRP1, ABCC1) and BCRP (ABCG2, placenta-specific ABC transporter, ABCP/breast cancer resistance protein, mitoxantrone resistance protein, MXR). MDR1 and MRP1 can transport a variety of hydrophobic drugs, and MRP1 can also extrude anionic drugs or drug conjugates. Additional members of the MRP/ABCC family have also been indicated to be involved in cancer multidrug resistance [for details, see [3]]. The transport properties of BCRP overlap both with that of MDR1 and the MRP type proteins, thus these three proteins form a special network involved with chemo-defense mechanisms.

Because of a significant role that ABC transporters play in cancer multidrug resistance and the body's protection against xenobiotics, sensitive and specific quantitative assays are required for the detection of the activity of these proteins. Also, higher throughput assay systems are required to screen for potential transporter-interacting partners. Estimation of the activity of ABC transporters is not easily achieved by routinely available classical non-functional methods, such as Northern blotting, RNase protection, RNA *in situ* hybridization, RT-PCR or immunostaining. ABC transporter protein expression is often not correlated with mRNA levels, as transcripts are often present below the detection threshold, since relatively few active transporter molecules can cause major alterations in drug transport. Additionally, functional activity of ABC transporters may not correlate with their expression levels determined by the methods listed above [4].

The ability of ABC transporters to actively transport compounds against the concentration gradient, across the cell membrane, has allowed the development of a number of functional assays to measure their level and function. Upon loading of the cells with lipophilic dye(s) capable of diffusing across cell membranes, the resulting fluorescence intensity of the cell(s) will depend upon the activity of the ABC transporters [5]. The cells with highly active transporters will display lower fluorescence intensity values because of the increased efflux of the dye/substrate. The functions of ABC transporters have been characterized by measuring the cellular uptake, efflux, or steady-state distribution of a number of fluorescent substrates using flow cytometry, fluorescence microscopy or fluorimetry. Substrate specificities of MDR1, MRP and BCRP transporters are distinct, but also overlapping [6].

Several drawbacks have been noted relating to the use of most fluorophores in ABC transporter activity assays arising from protein binding, dye sequestration, or changes in dye fluorescence intensity due to changes in intracellular parameters such as pH or free calcium levels [7]. To increase sensitivity of the method, hydrophobic ester derivatives, such as acetoxymethyl (AM) esters of the fluorescent dyes have been employed [8]. These cell permeable derivatives of fluorescent dyes may be actively exported from cells by MDR proteins. However, when these ester derivatives reach the cytosol, intracellular esterases cleave the ester groups rendering the export of resulting free dye by MDR proteins in the cells also possible [8], [9].

Clinicians are especially interested in identifying the drug resistance profile, substrate specificity and drug extrusion activity of the various multi-drug resistance proteins expressed in a given tumor sample. The demonstration of the transport activity of various multi-drug resistance proteins in the plasma membrane requires a sensitive and reproducible *in vitro* multi-drug resistance assay. To date, BCRP has gained prominence as one of the three major ATP-binding cassette (ABC) membrane efflux transporters, alongside MDR1 and MRP, conferring drug resistance in cancer and inflammation chemotherapies [10], [11], [12]. Besides being present in drug-resistant cancer and T-cells, BCRP is also endogenously expressed at a high level in human placenta and to a lesser extent in liver, small intestine, colon, ovary, veins, capillaries, kidney, adrenal, and lung, with little to no expression in brain, heart, stomach, prostate, spleen, and cervix [13]. It has been demonstrated that calcein AM is useful for the quantitative functional analysis of the presence of active *ABCB* and *ABCC* but not *ABCG* gene family member transporters in cells [11]. Thus, it is important to have a simple, reproducible and sensitive method to determine activity of all three types of ABC transporters in live cells.

To detect functional activity of drug efflux pumps, two assays using fluorescent dyes are commonly employed: one measures cellular dye accumulation and the other measures dye retention. The accumulation assay measures dye uptake in the presence or absence of known pump modulators. In the retention assay, the cells are loaded with the substrate in the absence of any reversal agent, washed, and then further incubated without dye but in the presence of reversing agents to allow time for the substrate to be transported out of the cell by a drug efflux pump. The distinction between accumulation and retention is necessary when evaluating cells for MDR phenotypes, as substrates act differently under the different experimental conditions. Accumulation/retention assays offer higher throughput, generic readouts (increase in fluorescence intensity), and are readily automated. However, these assays are not designed to distinguish MDR1 substrates from inhibitors [14] and do not directly measure transport.

Thus, there is need for a general non-toxic MDR probe that a) recognizes all three major ABC transporter's types in live cells (unlike the most common MDR probe calcein AM); b) is more sensitive than commonly used general MDR probes such as doxorubicin and mitoxantrone; c) can be used in the principal types of efflux assays discussed above; d) requires few processing steps and provides a reproducible protocol; e) is compatible with other common fluorescent dyes used in flow cytometry as well as diverse fluorescent proteins employed in cells.

## Materials and Methods

### Dyes and Reagents

eFluxx-ID® Green and Gold multidrug resistance detection kits were from ENZO Life Sciences, Inc. (Farmingdale, NY). Calcein AM, 3,3′-diethyloxacarbocyanine iodide [DiOC_2_(3)], chloromethylfluorescein diacetate (CMFDA) were obtained from Invitrogen Corp. (Carlsbad, CA). Mitoxantrone, doxorubicin, fumitremorgin C, penicillin, streptomycin, L-glutamine, HEPES, glucose, sodium piruvate, sodium bicarbonate were obtained from Sigma-Aldrich Chemical Company (St. Louis, MO). Eagle's Minimum Essential Medium with low glucose, F-12K medium, RPMI-1640 medium, McCoy's 5A Modified Medium, fetal bovine serum (FBS) and horse serum were obtained from American Tissue Culture Collection (ATCC, Manassas, VA). All components of eFluxx-ID® multidrug resistance detection kits were prepared according to a manufacturer's instructions. Additionally, stock solutions of the following dyes in DMSO were made: 1 mM (2000×) of calcein AM, 20 µg/ml of 3,3′-diethyloxacarbocyanine iodide [DiOC_2_(3), 1000×], 10 mM of pheophorbide A (2000×), 5 mM of chloromethylfluorescein diacetate (CMFDA, 1000×), 10 mM of mitoxantrone (1000×) and 5 mM of doxorubicin (1000×). Stock solutions of the dyes were aliquoted and stored at −20°C in the dark. The following stocks of additional inhibitors in DMSO were prepared: 5 mM of cyclosporin A (general specificity inhibitor [15], 1000×), 0.2 M of probenecid (MRP-specific inhibitor, 1000×), and 10 mM of fumitremorgin C (BCRP-specific inhibitor, 1000×). Mouse monoclonal [C219] antibody to P glycoprotein (ab3364), mouse monoclonal [QCRL1] antibody to MRP1 (ab3369), mouse monoclonal [MM0047-2J39] antibody to BCRP/ABCG2 (ab72788) and goat polyclonal secondary antibody to mouse IgG (FITC) (ab6785) were obtained from Abcam Inc. (Cambridge, MA). The isotype matching control antibodies (mouse monoclonal IgG2a antibody (ab18414) for [C219] and [MM0047-2J39]; mouse monoclonal IgG1 antibody (ab18448) for [QCRL1]) also were obtained from Abcam Inc.

### Cell lines

Human cervical adenocarcinoma epithelial cell line HeLa (putative MDR negative cell line), human lung carcinoma cell line A549 (expression of MPR1 and BCRP [16], [17]) and hamster ovary CHO K1 cell line (expression of all three major ABC transporter proteins [18], [19]) were obtained from ATCC (Manassas, VA). Human ileocecal colorectal carcinoma cell line HCT-8 (reported to express MDR1 and, to a lesser extent, MRP [20], [21]), human colorectal adenocarcinoma cell line HCT-15 (reported to over-express MDR1 [22]) and hepatocellular carcinoma Hep G2 (reported to express MRP [23], [24]) were also obtained from ATCC. Human acute promyelocytic leukemia cell lines HL-60 and HL-60/MX1 (mitoxantrone resistant derivative of the HL-60 cell line) were obtained from ATCC. All cell cultures were maintained in an incubator at 37°C, with 5% CO_2_ atmosphere in appropriate cell culture media as per ATCC recommendations.

### Cell viability assay

Cell viability was evaluated by a standard 3-(4,5-dimethylthiazol-2-yl)-2,5-diphenyltetrazolium bromide (MTT) viability assay, as described elsewhere [25]. Viability was calculated as a ratio of OD_595_ for treated cells to the OD_595_ of untreated control cells.

### MDR assay (dye uptake protocol)

Detailed protocol for the MDR assay is available in the Instruction Manual for eFluxx-ID® Green or Gold Multi-Drug Resistance Assay Kit (ENZO Life Sciences, Inc., Farmingdale, NY). Briefly, on the day of the assay, cells were collected, washed with PBS and incubated with or without MDR inhibitors in the presence of the eFluxx-ID® Green or Gold probes for 30 min at 37°C and analyzed immediately by flow cytometry. eFluxx-ID® Green or Gold probes are novel xanthene-based small molecule dyes developed for the detection of MDR activity in living cells. In order to exclude dead cells in the analyses, propidium iodide (PI) or 7-actinaminomycin D (7-AAD) solution was added to the cells during the last 5 min of incubation in some experiments.

To develop a minimal step assay procedure for the dye uptake protocol, three modifications of the protocol were investigated. One set of the cells was treated according to a general “no-wash” protocol (see Instruction Manual for the kit). In the second set of tubes, the reaction was stopped by rapid centrifugation (1 min) at relatively low speed (200×g). After washing with ice-cold PBS and discarding the supernatant, cells were re-suspended in 0.5 ml of ice-cold medium and kept on ice until flow cytometry analysis was performed. The third set of cells was simply spun down (1 min, 200×g) to remove the excess probe, resuspended in ice-cold medium and kept on ice until flow cytometry analysis was performed. eFluxx-ID® Green dye (ex/em 490/514 nm) fluorescence intensity was measured in the FL1/FITC (530/30 filter) channel, and eFluxx-ID® Gold dye (ex/em 530/555 nm) fluorescence intensity was monitored in the FL2/PE (585/42 filter) channel.

### Immunofluorescent flow cytometry MDR assay

Cells (5×10^5^ per sample) were collected by centrifugation (adherent cells were trypsinized first), washed with cold PBS, fixed with 0.25% paraformaldehyde (1 h at 2° to 8°C), permeabilized with 0.05% Tween 20 (15 min at 37°C), and incubated with 100 µL of corresponding primary antibody (5 µg/mL) overnight. Post-incubation, cells were washed twice with PBS/0.05% Tween 20, stained with secondary antibody, at 1∶5000 dilution, for 30 min at room temperature, washed again, re-suspended in PBS/2% FBS and analyzed using flow cytometry. Cells stained with isotype-matched antibody or with secondary antibody only were used as controls. Results were quantified by using Kolmogorov-Smirnov statistics [26] and by comparison of the mean fluorescence for each sample.

### Studies of the effect of intracellular pH and Ca^2+^/Mg^2+^ concentrations on dye signal

Cells were grown and prepared as described before. To study pH effect on MDR detection, different sets of tubes (with and without inhibitors) were prepared using cell culture medium titrated using 3 M potassium acetate (pH 5.0) or 1 M Tris (pH 8.0) to obtain solutions ranged from pH 5.5 to pH 8.0 (original, unmodified culture medium pH was 7.5). Cells were resuspended in medium of a certain pH value for 30 min at 37°C before the experiment. To study Ca^2+^/Mg^2+^ effect on the MDR detection, four experimental conditions were used: No Ca^2+^/Mg^2+^ (EDTA was added to culture medium to 5 mM final concentration); standard culture medium with Ca^2+^/Mg^2+^ (approximately 1 mM of each); high Ca^2+^ concentration medium (10 mM Ca^2+^/1 mM Mg^2+^) and high Mg^2+^ concentration medium (10 mM Mg^2+^/1 mM Ca^2+^). Cells were equilibrated in corresponding solutions for 30 min at 37°C, and all the following procedures were performed under described conditions.

### Flow cytometry

Experiments were performed using a FACS Calibur benchtop flow cytometer (BD Biosciences, San Jose, CA) equipped with a blue (488 nm) laser, and the signals were registered in the FITC (530/30 filter), PE (585/42 filter) and PerCP (670 LP filter) channels. eFluxx-ID® Green dye fluorescence was measured in the FL1/FITC channel, and eFluxx-ID® Gold dye fluorescence, in the FL2/PE channel. Data analysis was performed using FlowJo 8.8.2 software (Tree Star, Inc., Ashland, OR).

### Statistical analysis

All of the experiments were performed at least three times. Results were expressed as mean ± S.E. Statistical comparisons were made using an unpaired two-tailed Student's t-test. A P value of <0.05 was considered significant. Flow cytometry data were analyzed by comparison of median fluorescence, by using Kolmogorov-Smirnov statistics (D-value) [26] or by calculating MDR Activity Factor (MAF) values using the following formula: MAF = 100*((MFI_inh_−MFI_0_)/MFI_inh_), wherein MFI_inh_ and MFI_0_ are the mean fluorescence intensity values measured in the presence and absence of inhibitor [7].

## Results

### Demonstration of multidrug resistance in model mammalian cell lines using an MTT viability assay

**Figure 1:**
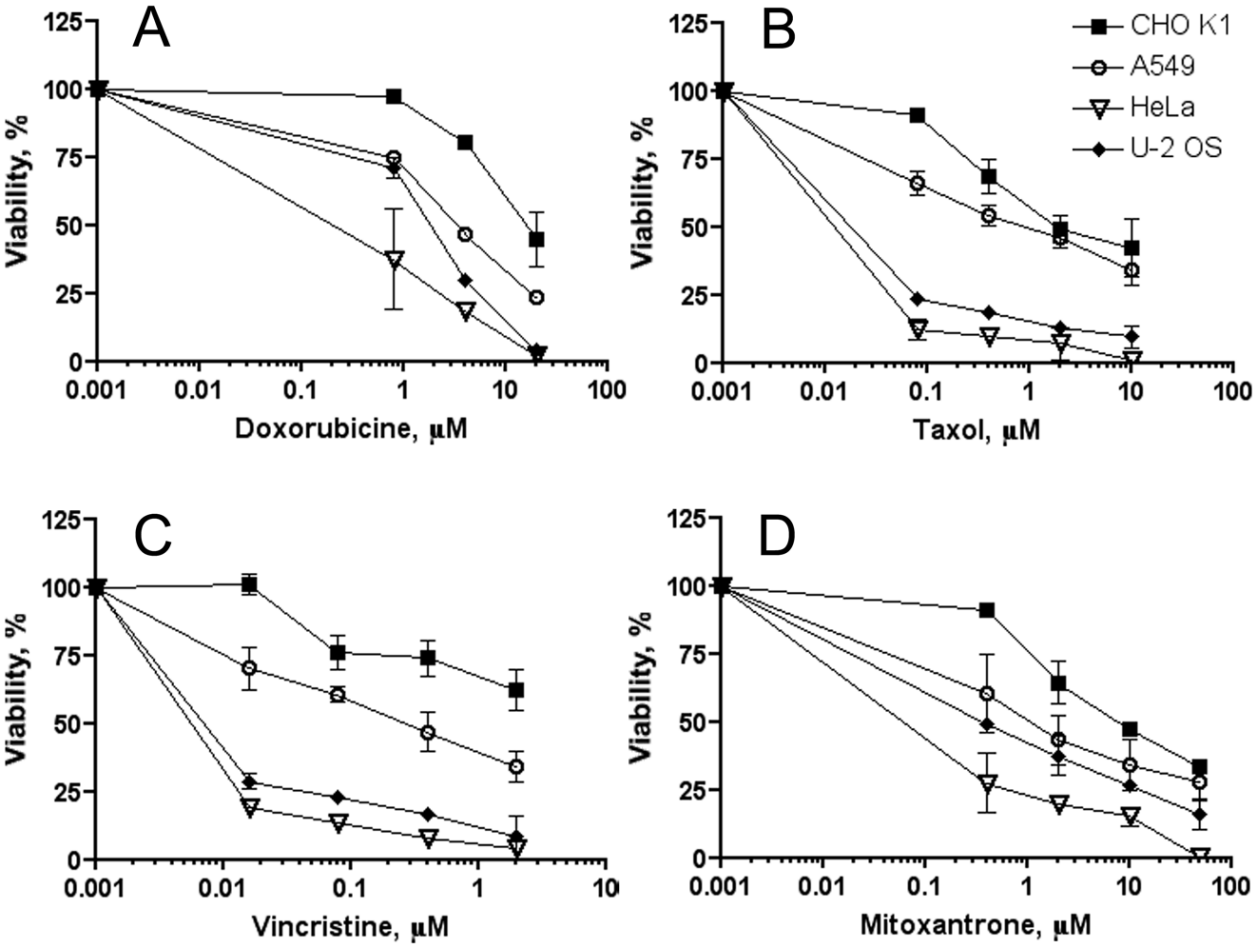
A–D. CHO K1 and A549 cell lines display increased chemoresistance toward the cytotoxic drugs that are mostly associated with MDR: doxorubicin (panel A), taxol (panel B), vincristine (panel C), and mitoxantrone (panel D). The U-2 OS cell line demonstrates moderately increased chemoresistance to a few drugs associated to MDR, such as doxorubicin (panel A) and mitoxantrone (panel D). The HeLa cell line was used as a non-chemoresistant control specimen. Cells were seeded in 96 well plates, treated with the different doses of indicated drugs, and a standard MTT viability test was performed 1 and 2 days post-treatment. Results are presented as a ratio of the OD_595_ of treated cells to the OD_595_ of the untreated cells.

To confirm drug resistance of the cell lines used as model systems in these studies, HeLa, U-2 OS, CHO K1 and A549 cells were seeded in 96-well plates at a density of 5×10^3^ cells/well and 24 h later, treated with increasing doses of different drugs that are commonly used for cancer chemotherapy and are known to be associated with the phenomenon of MDR (taxol, doxorubicin, vincristine, mitoxantrone). A standard MTT viability test was performed 24 and 48 h post-treatment, as described elsewhere [25]. Viability was calculated as the ratio of OD_595_ for treated cells to the OD_595_ of untreated control cells and presented as a function of a drug concentration (Figure 1A–D). Data from the MTT tests confirmed higher chemoresistance of CHO K1 and A549 cells compared to HeLa cells towards all toxic compounds used in the study (Fig. 1A–D). U-2 OS, CHO K1 and A549 cell lines were used as model cell lines demonstrating MDR activity in all subsequent studies.

### Demonstration of multidrug resistance in mammalian cell lines using immunostaining and flow cytometry

CHO K1, A549, HeLa, HCT-15, HepG2, HL-60 and HL-60/MX1 cells were collected, fixed and stained with the specific monoclonal antibodies against MDR1, MRP1 and BCRP transporters, as described in Materials and Methods. Cells were analyzed using flow cytometry, mean fluorescence values were registered for each sample and compared to the mean fluorescence of isotype antibody stained cells and cells stained with secondary fluorescence only. Population comparison was performed using FlowJo software and D-values (Kolmogorov-Smirnov statistical assay) were calculated for each sample (Table 1). A D-value greater than 0.2 was considered to be positive [26], [27], [28]. According to the results of the assay, CHO K1 and HCT-15 cell lines express somewhat higher levels of MDR1 (P-gp) protein than other cell lines tested. CHO K1, A549, HL-60, HL-60/MX1, HCT-15 and HepG2 cells are positive for MRP1 expression. CHO K1, A549 and HL-60/MX1 cell lines express BCRP.

**Table 1:** Detection of the expression levels of three major types of MDR proteins in cultured cell lines using immunostaining with monoclonal antibodies and a flow cytometry assay.

	Pgp Staining [C219]	MRP1 Staining [QCRL1]	BCRP/ABCG2 Staining[MM0047-2J39]
CHO K1	0.29±0.3	0.59±0.2	0.74±0.3
A549	0.12±0.02	0.63±0.3	0.57±0.2
HeLa	0.06±0.01	0.10±0.03	0.14±0.01
HL-60	0.13±0.07	0.22±0.07	0.17±0.09
HL-60/MX1	0.11±0.05	0.31±0.04	0.83±0.01
HCT-15	0.28±0.2	0.60±0.13	0.07±0.02
HepG2	0.18±0.05	0.58±0.06	0.14±0.001

Average D values (± SEM) for three representative experiments are presented.

### Development of a minimal step assay procedure for the dye uptake flow cytometry protocol

Preliminary experiments (MTT assays) with eFluxx-ID® Green and Gold probes demonstrated that both probes are not toxic to the cells (data not shown). To develop a minimal step assay procedure for the dye uptake protocol, three modifications of the protocol were investigated, as described in the Materials and Methods part. Stained cells were analyzed using flow cytometry. eFluxx-ID® Green dye (ex/em 490/514 nm) fluorescence intensity was measured in the FL1/FITC (530/30 filter) channel, and eFluxx-ID® Gold dye (ex/em 530/555 nm) fluorescence intensity was monitored in the FL2/PE (585/42 filter) channel. Mean fluorescence intensities (MFI) were registered for each probe used and MAF values were calculated for each sample. Comparison of MAF values showed no significant difference between the samples (data not shown). This observation indicates no wash step is required for performance of the assay.

Experiments with another model cell line (A549) and with the other tested probe (eFluxx-ID® Gold dye) demonstrated similar results, allowing one to conclude that in the novel MDR detection assay, the dye uptake protocol may be simplified and used without additional washing steps. However, in this case the samples must be analyzed within a 30 min interval of staining so the excess of the probe in the extracellular milieu would not affect the transport kinetics. If an immediate flow cytometry assay is not possible, then washing and storing on ice is recommended, since according to literature reports, most ABC transporters are inactive at 4°C [29], [30].

### Fluorescent signal does not vary with changes in intracellular pH or Ca^2+^ concentration

To study the effect of pH changes on MDR detection, CHO K1, A549 and HeLa cells were equilibrated in medium with different pH values (ranging from 5.5 to 8.0) as described in Materials and Methods, pretreated with inhibitors or left untreated and stained with calcein AM, eFluxx-ID® Green or Gold probes according to the above described dye uptake protocol, followed by flow cytometry assay. MAF values calculated for CHO K1 cells at various pH values were not significantly different for all three probes used in the assay (data not shown). For A549 cells, MAF values obtained at pH 5.5 were significantly different from the values obtained within the range of pH 6.0–8.0 for all three dyes. Uptake of all three dyes at pH 5.5 is also different from the uptake in the range of pH 6.0–8.0. For HeLa cells, MAF values obtained using eFluxx-ID® Green and Gold probes, were similar and did not show any significant activity of ABC transporters. By contrast, data obtained with calcein AM, showed activity of P-gp and MRP at the most extreme pH values of 5.5 and 8.0 (data not shown).

To investigate the influence of intracellular Ca^2+^/Mg^2+^ changes on MDR detection, cells were equilibrated in media with different concentrations of calcium and magnesium cations, as described in the Materials and Methods section and the dye uptake assay was performed with eFluxx-ID® Green and Gold probes as well as calcein AM probe (as a control) followed by flow cytometry analysis and MAF calculation for each experimental condition. Ten-fold excess of Ca^2+^ did not affect the MAF values obtained with all three dyes compared to the results obtained in standard media. Similarly, 10× excess of Mg^2+^ only slightly altered MAF values compared to the standard medium for all dyes used, but these changes could not be considered significant. It should be noted that the absence of Ca^2+^/Mg^2+^ in the medium significantly diminished MAF values for all three dyes. Overall, these experiments demonstrated that eFluxx-ID® Green and Gold probe fluorescence is pH-independent within a physiological range. Ca^2+^/Mg^2+^ concentrations (up to 10-fold above standard medium) also do not affect the results of the assay (data not shown).

### Confirmation of substrate specificity for eFluxx-ID® Green and Gold probes in mammalian cell lines using a flow cytometry dye uptake protocol

CHO K1, A549 and HeLa cells were grown on tissue culture dishes and on the day of assay were treated with eFluxx-ID® Green and Gold probes with and without inhibitors according to the described dye uptake protocol (Materials and Methods) and analyzed by flow cytometry. Calcein AM, DiOC_2_(3) (MDR1-specific probe, [31]], CMFDA [MRP-specific probe [24]) and pheophorbide A (BCRP-specific probe [32]) were used as additional control MDR probes with known substrate specificity. The specificity of the probes and MAF values obtained as a result of the test are listed in Table 2.

**Table 2:** Comparison of MDR activity detection in model cell lines using MDR probes and inhibitors of different specificity.

Cell Line	MDR Probe	Probe Specificity	Verapamil	Probenecid	Fumitremorgin C
CHO K1	Calcein AM	MDR1, MRP	63.9	55.3	6.4
	eFluxx-ID® Green		75.8	73.7	34.7
	eFluxx-ID® Gold		65.6	61.3	32.4
	DiOC_2_(3)	MDR1	86.6	2.4	0
	Pheophorbide A	BCRP	0.7	8.4	22.3
	CMFDA	MRP	34.3	56.9	0
	Calcein AM	MDR1, MRP	28.0	19.5	1.3
A549	eFluxx-ID® Green		16.1	38.7	33.7
	eFluxx-ID® Gold		12.3	23.1	31.5
	DiOC_2_(3)	MDR1	4.2	1.0	0.9
	Pheophorbide A	BCRP	4.0	14.2	36.0
	CMFDA	MRP	16.6	27.1	20.7
HeLa	Calcein AM	MDR1, MRP	13.0	12.2	0.2
	eFluxx-ID® Green		9.0	12.9	1.5
	eFluxx-ID® Gold		13.9	8.7	5.1
	DiOC_2_(3)	MDR1	0	0	0
	Pheophorbide A	BCRP	0	0.5	5.8
	CMFDA	MRP	7.6	9.0	11.4

Average MAF values for three representative experiments are provided, with SD not exceeding 10% for each value.

In CHO K1 cell line, MDR1 expression was detected by all specific dyes and by eFluxx-ID® probes; MRP expression was detected by all specific dyes and by both eFluxx-ID® probes; BCRP expression was detected by pheophorbide A, which is specific for this ABC transporter, and by eFluxx-ID® Green and Gold dyes. In A549 cell line, MDR1 expression was not detected by any specific dye, nor by either eFluxx-ID® probe; MRP expression was detected by all specific dyes and by eFluxx-ID® Green and Gold probes; BCRP expression was detected by pheophorbide A, and by eFluxx-ID® Green and Gold probes. In the HeLa cell line, no drug resistance was detected with any dye/inhibitor combination.

To confirm the specificity and selectivity of eFluxx-ID® probes further, we employed cultured cell lines that over-express a single transporter and are reported to exhibit multidrug resistance, HCT-15 (colorectal adenocarcinoma, overexpression of MDR1 [22]), HepG2 (hepatocellular carcinoma, overexpression of MRP [23], [24]), and HL-60/MX1 cell line (acute promielocytic leukemia, where mitoxantrone resistance is attributed to BCRP overexpression [33], [34]). Additionally, HCT-8 cell line reported to overexpress both MDR1 and MRP transporters [20], [21] was tested. HCT-8, HCT-15, HepG2, HL-60 and HL-60/MX1 cells were grown on tissue culture dishes and on the day of assay were incubated with eFluxx-ID® Green and Gold probes with and without inhibitors according to the described dye uptake protocol (Materials and Methods) and analyzed by flow cytometry. Calcein AM, DiOC_2_(3) (MDR1-specific probe, [31]), CMFDA (MRP-specific probe [24]) and pheophorbide A (BCRP-specific probe [32]) were used as additional control MDR probes with known substrate specificity. The specificity of the probes and MAF values obtained as a result of the test are listed in Table 3.

**Table 3:** Comparison of MDR activity detection in model cell lines overexpressing a single ABC transporter pump using MDR probes and inhibitors of different specificity.

Cell Line	MDR Probe	Probe Specificity	CsA	Ver	MK571	Nov
HCT-15	Calcein AM	MDR1, MRP	67.2±2.0	62.9±2.6	13.5±2.9	4.3±2.2
	eFluxx-ID® Green		66.2±7.0	64.4±6.7	21.1±2.6	17.1±3.7
	eFluxx-ID® Gold		68.5±6.6	65.8±6.0	16.2±2.6	16.6±1.7
	DiOC_2_(3)	MDR1	71.0±5.0	69.3±4.3	4.3±2.2	0
HepG2	Calcein AM	MDR1, MRP	59.0±2.4	22.0±2.9	40.7±4.5	3.0±0.1
	eFluxx-ID® Green		62.9±4.0	20.3±1.6	58.8±3.2	17.3±1.2
	eFluxx-ID® Gold		63.3±0.8	17.7±3.2	69.3±2.3	15.8±0.6
	CMFDA	MRP	50.4±1.3	12.8±1.3	52.2±3.8	7.0±1.7
HCT-8	Calcein AM	MDR1, MRP	46.5±5.1	25.9±0.9	31.1±4.7	7.1±0.4
	eFluxx-ID® Green		36.7±1.5	32.6±1.5	51.5±5.0	12.1±5.2
	eFluxx-ID® Gold		33.8±2.7	29.0±0.9	48.4±5.6	3.5±1.9
	DiOC_2_(3)	MDR1	24.7±0.9	19.1±3.7	9.8±1.2	0
	CMFDA	MRP	43.0±3.2	21.2±3.2	39.2±3.6	0
HL-60	Calcein AM	MDR1, MRP	15.2±2.5	2.5±1.1	8.0±1.7	8.6±1.5
	eFluxx-ID® Green		11.8±2.8	5.2±0.6	19.9±2.9	8.5±3.1
	eFluxx-ID® Gold		0.29±0.3	14.0±3.4	15.8±0.6	7.2±1.6
	Pheophorbide A	BCRP	6.5±1.7	6.8±0.6	7.6±1.6	8.5±1.4
HL-60/MX1	Calcein AM	MDR1, MRP	18.2±2.7	1.6±0.3	10.8±1.8	5.9±1.2
	eFluxx-ID® Green		33.1±3.4	17.8±8.3	19.0±1.9	37.5±2.6
	eFluxx-ID® Gold		30.2±3.4	14.6±2.9	16.1±3.8	24.6±3.9
	Pheophorbide A	BCRP	22.5±3.0	7.6±3.8	10.2±3.7	23.4±0.8

Average MAF values + SEM for three representative experiments are provided. Negative MAF values have been replaced with 0.

In HCT-15 cell line, MDR1 expression was detected by all probes used, when cyclosporin A or verapamil were added. In contrast, MK571 inclusion does not affect fluorescence of either eFluxx-ID® probe or calcein AM, confirming the absence of active MRP type transporters in the cell line. In the HepG2 cell line, MDR1 expression was not detected by any probe. Instead, a significant MRP expression was detected by all probes used, including CMFDA, which is specific for MRP type pumps. BCRP expression was not detected in either cell line, as shown in the Table 2B (in the presence of novobiocin). In the HL-60/MX1 cell line, significant fluorescence increase of both eFluxx-ID® probes and pheophorbide A probe was observed in the presence of novobiocin and fumitremorgin C (specific BCRP inhibitors). No drug resistance was detected with any other dye/inhibitor combination. Calcein AM fluorescence was not changed by the treatment. In contrast, parental HL-60 cells do not exhibit specific BCRP activity or any other ABC transporter activity.

### Profiling ABC transporters using eFluxx-ID® Green and Gold dyes in conjunction with various other specific MDR probes

CHO K1 cells were grown in tissue culture dishes and on the day of assay were treated according to a dye uptake protocol and analyzed by flow cytometry as described in Materials and Methods. eFluxx-ID® Green and Gold probes, DiOC_2_(3) (MDR1-specific probe, [31]), CMFDA (MRP-specific probe [24]) or pheophorbide A (BCRP-specific probe [32]) were added to the cells alone or in combination (general dye + specific dye or general dye + two specific dyes). Dye combinations were selected to facilitate spectral separation of the fluorescence emission profiles. The concentrations of the probes are specified in the Materials and Methods section.

Following 30 min incubation at 37°C, cells were analyzed by flow cytometry. Cell population profiles were collected directly without any compensation correction. Additionally, double or triple stained samples were also collected after electronic compensation correction using singly stained samples.

Pheophorbide A, which is a specific probe for BCRP, was used in combination with either green (eFluxx-ID® Green) or orange (eFluxx-ID® Gold) MDR general probes. In BCRP-expressing CHO K1 cells, fluorescence decreased in both channels (FL1/FITC and FL3/PerCP or FL2/PE and FL3/PerCP) if cyclosporin A (general MDR inhibitor [15]) or fumitremorgin C (specific BCRP inhibitor [35]) were not present (Table 4). Quantitative characteristics of the changes in fluorescence (expressed as D- or MAF values), corresponded to values obtained with single probes. It is important to note that in the case of dual probe treatment with eFluxx-ID® Gold dye, compensation corrections were required to minimize overlap with the other dye spectra. In the cells that were BCRP-negative but positive for other types of ABC transporters, red fluorescence intensity was high, while green or orange fluorescence was dim.

**Table 4:** Examples of multiplex (dual) probe treatment for MDR activity profiling in the CHO K1 cell line.

Probes	Detection Channel	D-values Post Inhibitor Treatment(compared to uninhibited cells			
		CsA	Verapamil	MK-571	FTC
eFluxx-ID® Green	FITC	0.94	0.9	0.98	0.56
Pheophorbide A	PerCP	0.25	0.14	0.15	0.33
eFluxx-ID® Gold	PE	0.89	0.94	0.96	0.51
Pheophorbide A	PerCP	0.25	0.11	0.14	0.34
eFluxx-ID® Gold	PE	0.84	0.82	0.96	0.44
DiOC_2_(3)	FITC	0.94	0.96	0.13	0.16
eFluxx-ID® Gold	PE	0.9	0.94	0.85	0.53
CMFDA	FITC	0.49	0.1	0.91	0.06

(Average D values for three representative experiments are provided as characteristics of MDR, with SD not exceeding 10% for each value).

If DiOC_2_(3) was used in combination with eFluxx-ID® Gold dye, in MDR1 expressing cells, both dyes are pumped out in the absence of the inhibitors and cells exhibit decreased fluorescence in both the FL1/FITC and FL2/PE channels. In MRP or BCRP expressing cells, only eFluxx-ID® Gold dye was pumped out in the absence of the inhibitors, and cells showed lower fluorescence in the FL2/PE but not FL1/FITC channel. According to these considerations, when both dyes were used in the CHO K1 cell line, orange fluorescence was bright in all cases when cells were treated with the inhibitors, but green fluorescence was bright only in the presence of verapamil (Table 4).

CMFDA could also be used in combination with eFluxx-ID® Gold dye. In MRP expressing cells, both dyes were pumped out of the cells in the absence of the inhibitors and cells demonstrated a decreased fluorescence in both the FL1/FITC and FL2/PE channels. In MDR1 or BCRP expressing cells, only the eFluxx-ID™ Gold was pumped out and fluorescence declined in the FL2/PE but not FL1/FITC channel. Accordingly, in CHO K1 cells, orange fluorescence was sustained as a bright signal when specific inhibitors were used, but green fluorescence remained bright only when the MRP inhibitor (MK-571) was applied.

For triplex probe profiling, eFluxx-ID® Gold dye was employed as a general specificity probe, and DiOC_2_(3), CMFDA and pheophorbide A were employed as specific probes (Table 5).

**Table 5:** Examples of multiplex (triple) probe treatment for MDR activity profiling in the CHO K1 cell line.

Probes	Detection Channel	D-values Post Inhibitor Treatment(compared to uninhibited cells			
		CsA	Verapamil	MK-571	FTC
DiOC_2_(3)	FITC	0.98	0.98	0.13	0.11
eFluxx-ID® Gold	PE	0.81	0.91	0.97	0.54
Pheophorbide A	PerCP	0.4	0.17	0.13	0.41
CMFDA	FITC	0.47	0.10	0.89	0.14
eFluxx-ID® Gold	PE	0.93	0.97	0.95	0.64
Pheophorbide A	PerCP	0.20	0.18	0.09	0.27

(Average D values for three representative experiments are provided as characteristics of MDR, with SD not exceeding 10% for each value).

When DiOC_2_(3) and pheophorbide A were used in CHO K1 cell line in conjunction with eFluxx-ID® Gold dye, orange (FL2/PE) fluorescence remained bright after treatment with all inhibitors used for the experiment. Green (FL1/FITC) fluorescence decreased unless cyclosporin A (general MDR inhibitor) or verapamil (MDR1 inhibitor) was present. Dark red (FL3/PerCP) fluorescence remained bright only when cyclosporine A (general MDR inhibitor) or fumitremorgin C (BCRP inhibitor) were present.

Similarly, with the combination of eFluxx-ID® Gold dye, CMFDA and pheophorbide A, orange (FL2/PE) fluorescence remained bright after treatment with all inhibitors used in the experiment. Green (FL1/FITC) fluorescence decreased unless cyclosporin A (general MDR inhibitor) or MK-571 (MRP inhibitor) was present. Dark red (FL3/PerCP) fluorescence remained bright only when cyclosporin A (general MDR inhibitor) or fumitremorgin C (BCRP inhibitor) were present.

### eFluxx-ID® Green and Gold probes are more sensitive for MDR activity detection than other commonly used probes of general specificity

**Figure 2:**
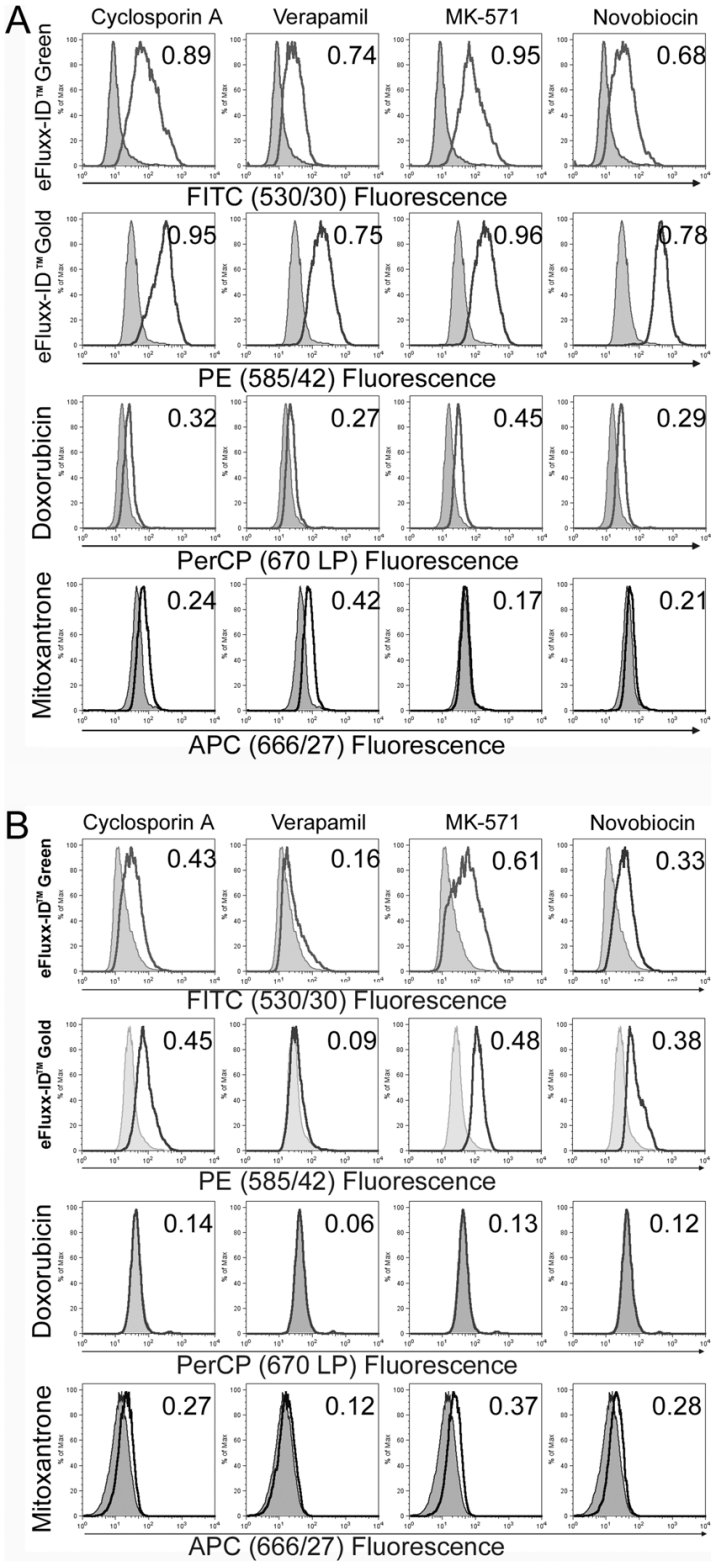
eFluxx-ID® MDR probes detect all three major types of ABC transporters in a similar manner as doxorubicin and mitoxantrone probes, but are significantly brighter, providing much higher sensitivity compared with the other dyes. Model cell lines (CHO K1, panel A, and A549, panel B) were trypsinized, washed with PBS, aliquoted at 5×10^5^ cells/sample, and treated in triplicates with different inhibitors (5 µM of cyclosporin A, 20 µM of verapamil, 50 µM of MK-571, or 0.05 µM of novobiocin) or left untreated. Tested probes (eFluxx-ID® Green, eFluxx-ID® Gold dyes, doxorubicin or mitoxantrone) were added to every sample. The cells were incubated with the dye(s) in the presence or absence of inhibitors for 30 min at 37°C. Then cells were immediately analyzed by flow cytometry. Population comparison was performed using Kolmogorov-Smirnov statistics [26]. Clear histograms represent sample fluorescence in the presence of the inhibitor, shaded – without the inhibitor. The numbers indicate average D-values for each sample from at least three independent experiments, with SD not exceeding 10% for each value.

Sensitivity and specificity of eFluxx-ID® Green and Gold probes, mitoxantrone and doxorubicin were analyzed and compared using three model cell lines (CHO K1, A549 and HeLa). Cells were grown in tissue culture dishes and on the day of assay were treated according to a dye uptake protocol and analyzed by flow cytometry, as described in Materials and Methods. The results of the assay are presented in Figure 2 (representative experiment) where average D-values from three independent experiments are displayed. All four probes were capable of detecting all three major types of ABC transporters, however the eFluxx-ID® Green and Gold probes generate substantially higher fluorescence intensity (as shown by D-values), demonstrating they are capable of providing more sensitive detection of MDR.

### Determination of EC_50_ Inhibitor Values Using eFluxx-ID® Green and Gold probes and flow cytometry

**Figure 3:**
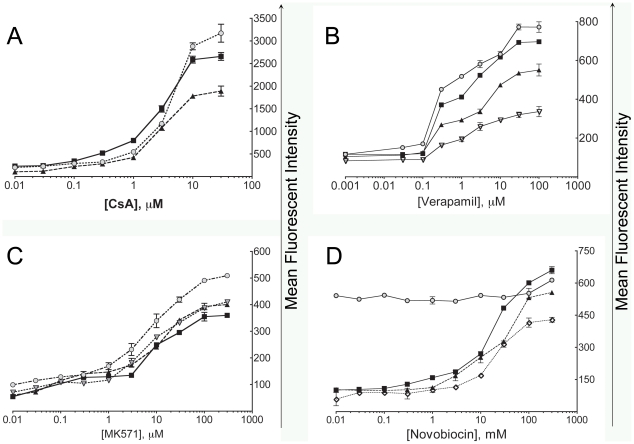
Concentration-dependent inhibitory effect of various general and specific inhibitors on MDR probes accumulation in CHO K1 (panel A), HCT-15 (panel B), HepG2 (panel C) and HL-60/MX1 (panel D) cells. The following MDR probes were used: eFluxx-ID® Green (filled squares), eFluxx® Gold (filled triangles), calcein AM (grey circles), DiOC_2_(3) (open triangles), CMFDA (open diamonds), pheophorbide A (filled diamonds). Cells were stained with the indicated probes in the presence of the various concentrations of the appropriate inhibitors. EC_50_ values are defined as the concentration of the inhibitor resulting in half-maximum inhibition of dye accumulation.

CHO K1 cells were grown in tissue culture dishes and on the day of assay were pre-treated with different concentrations of cyclosporin A (general MDR inhibitor) or left untreated. Then, cell samples were stained with eFluxx-ID® Green or Gold probes and analyzed using flow cytometry. Recorded MFI were plotted against CsA concentrations (Figure 3A), and EC_50_ (dose at which 50% of multidrug resistance activity is inhibited) was calculated. The results estimated an EC_50_ value for CsA of 2 µM when eFluxx-ID® Green probe was used and 2.7 µM when the Gold probe was used. The EC_50_ value obtained using calcein AM was 3 µM. These data agree well with the literature data for inhibitory activity of cyclosporin A [36].

Similar EC_50_ detection experiments were performed using cell lines overexpressing a single transporter and a corresponding specific inhibitor. We determined EC_50_ of verapamil using HCT-15 cell line, EC_50_ of MK571 using HepG2 cell line and EC_50_ of novobiocin and fumitremorgin C using HL-60 and HL-60/MX1 cell lines. Fluorescent probes specific for each particular transporter and calcein AM were employed for comparison. The results are presented in Figure 3, panels B–D. For verapamil, the EC_50_ values obtained using HCT-15 cell lines were 0.22 µM and 0.24 µM (eFluxx®-ID Green and Gold respectively), and 0.2 µM for both calcein AM and DiOC_2_(3) (Figure 3B). EC_50_ values for MK571 obtained using HepG2 cell line were as follows: 5 µM for both eFluxx®-ID Green and Gold, 3.7 µM for CMDFA and 3 µM for calcein AM (Figure 3C). Novobiocin EC_50_ values were determined using HL-60/MX1 cell lines as 10.2 µM (eFluxx®-ID Green), 10.3 µM (eFluxx®-ID Gold) and 10.1 µM (pheophorbide A). Calcein AM can not be used for detection of EC_50_ values of BCRP inhibitors in similar systems (Figure 3D). In summary, Figure 3 demonstrates high concordance of the data obtained using probes known to be sensitive and specific to the assayed MDR transporters.

### Compatibility of eFluxx-ID® Green and Gold probes with propidium iodide (PI) and 7-AAD, the most common dead cell indicator dyes

CHO K1 cells were grown in tissue culture dishes and on the day of assay were treated according to the dye uptake protocol described in Materials and Methods. After 30 min incubation with probe, in the presence or absence of various inhibitors, PI was added to each sample to a final concentration of 1 µg/mL and samples were analyzed by flow cytometry as described in Materials and Methods. Single stained samples (PI only, eFluxx-ID® Green dye only and eFluxx-ID® Gold dye only) were analyzed to validate data obtained with the two dye combinations. Data were collected uncompensated. For comparison, compensation corrections were performed using unstained cells, PI only stained dead cells and eFluxx-ID® Green or Gold dye stained cells (in the presence of cyclosporin A or MK-571 which results in a bright fluorescent signal).

Data presented in Table 4, clearly demonstrate that use of PI does not affect MDR detection when eFluxx-ID® Green and Gold dyes are used. In turn, green or orange fluorescence resulting from the MDR detection does not affect determination of the number of dead cells in the sample. In certain cases (when the combination of eFluxx-ID® Gold dye and PI is used, and inhibitor treatment leads to a significant orange fluorescence intensity increase), compensation corrections are required in order to avoid overestimation of dead cell staining. Similar results were obtained when 7-AAD, another common viability stain was used in the experiments (data not shown).

### Multiplexed MDR detection by flow cytometry in U-2 OS cells expressing green fluorescent protein (GFP) or red fluorescent protein (RFP)

U-2 OS parental cells and U-2 OS cells expressing an RFP-tagged mitochondrial targeting sequence of human cytochrome *c* oxidase subunit VIII precursor (Marinpharm GmbH, Luckenwalde, Germany) were treated with or without inhibitors and stained with eFluxx-ID® Green dye. Comparable cells expressing a GFP-tagged construct were stained with eFluxx-ID® Gold dye, as described in the Materials and Methods. To exclude dead cells, PI solution was added to the cells during the last 5 min of incubation (final concentration was 1 µg/mL). Single stained samples of parental U-2 OS cells were prepared with each dye for compensation correction.

Experiments conducted with GFP-expressing U-2 OS cells stained with eFluxx-ID® Gold probe and PI demonstrated feasibility of MDR activity detection in such systems (Table 5A and 5B). However, compensation corrections were necessary for accurate estimation of PI positive cells. PI fluorescence (red) does not interfere significantly with green and orange dye signal and green signal does not affect red fluorescence, either. However, orange fluorescence does affect PI measurements significantly (particularly, when the inhibition of MDR was observed and orange signal increased significantly). Thus, those samples stained with eFluxx-ID® Gold dye and PI required electronic compensation correction. Based upon these results, recommendations are to use single stained samples for each dye as well as unstained cells in order to define appropriate electronic compensation settings. Nevertheless, results obtained for the MDR activity do not depend upon compensation correction in all cases.

## Discussion

We describe two novel probes for functional detection of multidrug resistant phenotypes in live cells (both suspension and adherent) by measuring active dye efflux mediated by different ABC transporter proteins using flow cytometry. The method involves exposing cells of a biological specimen to a novel xanthene-based small molecule probe (either eFluxx-ID® Green or Gold dye) and measuring the amount of fluorescence that accumulates in the specimen cells compared to control cells (cells that do not exhibit MDR or cells that have inhibited MDR activity). Reduced fluorescence accumulation in tested cells correlates with multidrug-resistance. Using specific inhibitors of ABC transporters along with the probe allows easy identification of even small degrees of functional efflux, as well as definition of the type of multidrug resistance present in the specimen.

No genetically modified cell lines were used as model systems in the present study, since the levels of MDR activity in these modified cell lines is much higher than those encountered with standard clinical samples [4]. Instead, we used a few well-characterized cell lines as model systems demonstrating MDR activity in all subsequent studies. The chemoresistance of the investigated cell lines has previously been reported elsewhere [16]–[24], [33], [34]. Additionally, we performed the MTT viability assay with CHO K1, A549 and HeLa cell lines to determine IC_50_ of several drugs and to demonstrate their multidrug resistance profiles. In parallel, immunostaining flow cytometry was employed to detect levels of specific multidrug resistant proteins in cell lines used in the study. In general, results of both assays correlated with the data obtained by using e-Fluxx-ID® probes (and other commonly used probes for flow cytometry-based MDR detection). There are a few discrepancies between immunostaining and fluorescent probe uptake methods. For example, immunostaining detects quite low levels of MDR1 protein in CHO K1 cell line, while these cells demonstrated high MDR1 activity in the uptake assay with calcein AM, DiOC_2_(3) and both eFluxx-ID® probes. One of the possible reasons for this is that the physiological expression level of this transporter is quite close to the detection threshold of the antibody. Additionally, relatively small changes in MDR1 expression levels could cause major alterations in drug transport activity. Another discrepancy between the immunohistochemistry and functional assay results was the relatively high levels of MRP1 expression in HCT-15 cell line detected with antibody staining, in contrast none of the probes (calcein AM, eFluxx-ID® probes, CMFDA) were able to detect MRP activity in this cell line. A possible explanation for these results is that in this particular case, the high protein expression levels did not ensure high activity of the pump (the threshold of an antibody detection is much higher than the levels of the protein expression that is necessary to cause changes in the drug transport). The fact that this particular antibody demonstrated quite high levels of MRP1 staining in all tested cell lines may serve as indirect proof of this statement.

We confirmed the specificity and selectivity of eFluxx-ID® probes using several experimental approaches. First, we compared the new probes with the commonly used classical probes of known specificity in conjunction with particular specific MDR inhibitors (Table 2). Although verapamil is known to inhibit MRP pumps at higher concentrations [36], we used low concentrations of verapamil that make it more specific for MDR1. Additionally, experiments with MDR-specific inhibitors (MK571 and probenecid) were performed to confirm the specificity of inhibition. As demonstrated in Table 2, eFluxx-ID® and calcein AM dyes detect similar MDR1 and MRP levels in CHO K1 cells, and detect only MRP but not MDR1 in A549 cells. In contrast to calcein AM, which is not able to detect BCRP activity, eFluxx-ID® probes detect significant BCRP activity in both CHO K1 and A549 cell lines. Results obtained with eFluxx-ID® probes corroborate the MTT chemoresistance data (Figure 1A–D) and agree with the published literature [17], [18], [19]. For further validation, we employed cultured cell lines that over-express a single transporter and are reported to exhibit multidrug resistance, HCT-15 ([colorectal adenocarcinoma, overexpression of MDR1 [22]), HepG2 (hepatocellular carcinoma, overexpression of MRP [23], [24]), and HL-60/MX1 cell line (acute promielocytic leukemia, presumed overexpression of BCRP since mitoxantrone resistance is usually attributed to BCRP overexpression [33]). Additionally, the HCT-8 cell line, reported to overexpress both MDR1 and MRP transporters [20], [21], was tested. Again, specific inhibitors were utilized, and classical probes with known specificities were used for the control treatments (Table 3). The data presented in this table, were corroborated by the data obtained with an alternative method of detection of MDR protein expression (Table 1A). Quantitatively, the results of immunostaining generally demonstrate good correlation with the results of the functional uptake assays. The comparison of the data presented in both tables confirmed that both eFluxx-ID® probes can specifically detect a particular MDR activity when used together with a corresponding modulator of ABC transporter activity.

The sensitivity of the developed probes is comparable to the most sensitive commonly used classical MDR detection probes (like calcein AM, DiOC_2_(3), CMDFA). EC_50_ values for various specific inhibitors detected using eFluxx-ID® probes and cell lines expressing a single ABC transporter are very close to the EC_50_ values detected using the commonly used probes (Figure 3). From another perspective, unlike the most commonly used classical MDR probes, eFluxx-ID® MDR probes are capable of detecting and distinguishing between all three of the major types of ABC transporters in live cells: MDR1 (P-glycoprotein), MRP and BCRP. Importantly, new draft guidelines from the United States Food and Drug Administration (FDA) now recommend providing *in vitro* drug interaction information on drug efflux transporters, particularly MDR1 and BCRP, for New Drug Application (NDA) filings [37]. Calcein AM is only capable of measuring one of these two key drug efflux transporters [6], [11], while the eFluxx-ID® probes can simultaneously monitor both.

Using eFluxx-ID® MDR probes offers a rapid, reproducible, specific and relatively simple way for functional detection of ABC transporter proteins that allows high-volume specimen throughput and employs widely available instrumentation. The previously described classical dye-based uptake protocols include several washing steps, thus being time- and effort consuming. It is demonstrated that the washing steps are optional when employing eFluxx-ID® probes and may be omitted from the uptake protocol to simplify the assay and make it more user-friendly. However, if large numbers of samples (over ∼60) are to be analyzed simultaneously, the excess dye should be washed away and samples kept on ice until the measurement is performed. Otherwise, samples would have to be analyzed in small batches to avoid measurement artifacts. The described assay allows quantification and comparison of multidrug resistance levels between different samples or cell lines (by calculating MAF values or using Kolmogorov-Smirnov statistics). eFluxx-ID® probes demonstrate fast internalization, favorable uptake/efflux kinetics and much better sensitivity than the commonly used fluorescent substrates of general specificity, such as doxorubicin and mitoxantrone (Figure 2).

eFluxx-ID® Green and Gold probes demonstrate performance similar to or better than calcein AM with respect to pH independence of signal and can be used in an operational range of pH 6.0 through 8.0. Differences in fluorescence intensities of the eFluxx-ID® MDR probes in the described protocol are solely dependent upon the presence of the ABC transporters and do not depend upon the activity of cytosolic esterases, pH and intracellular Ca^2+^ concentration (similarly to calcein AM dye, which calcein-AM is not fluorescent and free calcein is not a substrate of the multidrug transporter) [7], [8].

The 488 nm laser excitable eFluxx-ID® MDR probes are compatible with a wide range of instruments, and with other common fluorescent dyes/fluorescent proteins typically used in flow cytometry. With appropriate compensation correction, both probes can be used in combination with PI or 7-AAD, the most common cell viability dyes employed in flow cytometry. Moreover, their spectral characteristics are compatible with common dyes used in multicolor flow cytometry experiments (such as PE), and with different fluorescent proteins such as GFP and RFP. Additionally, the developed MDR probes can be used in conjunction with other specific MDR probes (with compatible spectral characteristics) to facilitate the discrimination of the type of multidrug resistance in a cell (Tables 4 and 5).
